# Human resource development and antiretroviral treatment in Free State province, South Africa

**DOI:** 10.1186/1478-4491-6-15

**Published:** 2008-07-28

**Authors:** Dingie HCJ van Rensburg, Francois Steyn, Helen Schneider, Les Loffstadt

**Affiliations:** 1 Centre for Health Systems Research & Development, University of Free State, South Africa; 2 Centre for Health Systems Research & Development, University of Free State, South Africa; 3 Centre for Health Policy, University of Witwatersrand and Centre for Health Systems Research & Development, University of Free State, South Africa; 4 Directorate: Human Resources, Free State Department of Health, South Africa

## Abstract

**Background:**

In common with other developing countries, South Africa's public health system is characterised by human resource shortfalls. These are likely to be exacerbated by the escalating demand for HIV care and a large-scale antiretroviral therapy (ART) programme. Focusing on professional nurses, the main front-line providers of primary health care in South Africa, we studied patterns of planning, recruitment, training and task allocation associated with an expanding ART programme in the districts of one province, the Free State.

**Methods:**

Data collection included an audit of professional nurse posts created and filled following the introduction of the ART programme, repeated surveys of facilities providing ART over two years to assess the deployment of staff, and secondary data analysis of government personnel databases to track broader patterns of recruitment and training.

**Results:**

Although a substantial number of new professional nurse posts were established for the ART programme in the Free State, nearly 80% of these posts were filled by nurses transferring from other programmes within the same facility or from facilities within the same district, rather than by new recruits. From the beginning, ART nurse posts tended to be graded at a senior level, and later, in an effort to recruit professional nurses for the ART programme, the majority (54.6%) of nurses entering the programme were promoted to a senior level. The vacancy rate of nurse ART posts was significantly lower than that of other posts in the primary health care (PHC) system (15.7% vs 37.1%). Nursing posts in urban ART facilities were more easily filled than those in rural areas, exacerbating existing imbalances. The shift of nurses into the ART programme was partially compensated for by the appointment of additional support staff, task shifting to community health workers, and a large investment in training of PHC workers. However, the use of less-trained, mid-level enrolled nurses and nursing assistants in the ART programme remained low.

**Conclusion:**

The introduction of the ART programme has revealed both strengths and weaknesses of human resource development in one province of South Africa. Without concerted efforts to increase the supply of key health professionals, accompanied by changes in the deployment of health workers, the core goals of the ART programme – i.e. providing universal access to ART and strengthening the health system – will not be achieved.

## Background

Shortages of trained health personnel represent nothing less than a crisis of epidemic proportions in the developing world. In some of the poorest countries, health systems are in danger of collapse due to the lack of health staff to deliver services [1]. The global gap in the supply of health workers is estimated to be 4.3 million, with 57 countries depicted as 'countries with critical shortage' experiencing shortfalls of 2.4 million doctors, nurses and midwives. Human resource challenges manifest not only in shortages of health workers, but also in disparities in their distribution, poor training capacity, skills and skills mix deficits, and weak management and supervisory systems [2]. To aggravate matters, HIV and AIDS (even more so labour-intensive ART) are superimposing cumulative burdens on already overstretched health systems of those countries at the epicentre of the epidemic. Despite these challenges, human resource management systems remain weak and fragmented in most countries [1-6].

The high demand for human resources for health applies also to South Africa, especially against the backdrop of a still growing HIV/AIDS epidemic and, since 2003, the introduction of a massive and expanding public sector ART programme. With more than five million South Africans infected, the 'Comprehensive Plan' intends to provide ART access to more than a million people by 2008 [7]. In addition to reducing morbidity and mortality from HIV/AIDS, a central aim of the Comprehensive Plan is "to strengthen the national health care system overall" [6]. In human resource terms, such systems strengthening is foreseen through the injection of large numbers of additional health care staff into the public health workforce, including 975 doctors, 2924 professional nurses, 661 pharmacists, 526 dieticians and 488 social workers by March 2008 [7].

Concerns have been raised, however, as to where the large numbers of additional health professionals needed for public sector ART will come from in light of the international brain drain, unequal geographical and sectoral distributions of the health workforce, and high vacancy and turnover rates in the public health sector [8-12]. South Africa currently has fewer public health workers, including professional nurses, than it did ten years ago, both in absolute numbers and relative to population size [11,13]. To aggravate shortages, South Africa's national health system is structurally characterised by a deep public-private sector divide. In terms of human resources, this is reflected in large inequalities in the distribution of health personnel. In the case of professional nurses, in 2005, of the 99 534 professional nurses registered with the South African Nursing Council only 43 660 (43.9%) were employed in the public sector, despite the fact that this sector covers more than 80% of the population [10]. Against this backdrop, some have gone so far as to suggest that a badly managed introduction of ART "could do more harm than good" [14], and that the shortage of skilled personnel and inequities in human resource provisioning in the public sector might worsen, especially in the primary health care (PHC) system and particularly in poorer and rural districts.

Calls are thus for ART scale-up to go hand-in-hand with comprehensive health systems development, upgrading of human resource capacity and realistic targets [15,16]. To accomplish this, policy makers and managers in high-burden countries have to devise new strategies and even make paradigm shifts to cope with human resource challenges at regional, national, sub-national and facility levels, either by exploring new human capacity or by utilising and managing the existing pools of personnel better [4,17-19]. Apart from staff retention and increased production strategies [2], a common response to human resource shortages in poor-resourced settings is the use of substitute health workers, i.e. mid-level, support and lay/community health workers, to relieve pressures on professional staff. The concepts of 'task shifting' and 'task delegation' are commonly used in reference to this response. Four such forms in African countries have been described: (1) indirect substitution or delegation of tasks to an existing but different profession (e.g. from doctors to nurses or pharmacists); (2) direct substitution or delegation of tasks from professionals to less-trained, mid-level health workers within the same profession (e.g. using nursing assistants in lieu of professional nurses); (3) delegating non-technical tasks to lower-trained (even lay) cadres of staff (e.g. employing administrative clerks and community health workers [CHWs] to support nurses); and (4) intra-cadre skills delegation or shifting of tasks to less-trained cadres from the same profession (e.g. from medical specialists to general practitioners) [3].

After three years of programme expansion in South Africa, it is necessary to ask whether and how, firstly, the health system has thus far mobilised the essential human resources for ART, and, secondly, the extent to which the aim of overall system strengthening through the ART programme is being achieved. This paper reports on aspects of the human resource dynamics following the inception in 2004 of a public sector ART programme in one province (Free State) of South Africa. In particular, it examines whether the stated goal of strengthening human resource capacity through the establishment of extra posts, appointment of extra personnel, and the enhanced training of staff was achieved or in fact undermined. Core human resource management shortfalls and challenges and strategies to address these are highlighted.

## Methods

Facilities providing ART in the Free State comprise three types: treatment sites (referral hospitals), assessment sites (referring PHC clinics) and combined treatment-assessment sites (either community health centres or larger PHC clinics, which fulfil both assessment and treatment functions). The latter types were introduced in predominantly sparsely populated rural/small-town areas. PHC nurses assess patients for ART at assessment and combined sites, and patients who meet baseline criteria are referred to treatment sites (or doctors at the combined sites) where they are certified for ART by an ART-trained doctor [12,20]. During Phase I of ART roll-out in the province (May to December 2004), 4 treatment sites, 13 assessment sites and 3 combined sites were activated. In Phase II (2005/2006), a further 3 treatment, 8 assessment and 7 combined sites were introduced.

The research reported here forms part of a larger, longitudinal evaluation of the roll-out of public sector ART in the Free State. The paper focuses on issues pertaining to staffing of the programme with professional nurses – regarded as the backbone of PHC in South Africa – in the first 38 ART sites of the five districts in the province.

The study tracked and reconstructed human resource dynamics over a period of almost three years through assessments of the following:

1. New staffing establishments created and recruitment patterns in the inception phases of the ART programme, in a staffing audit conducted in November 2004, i.e. after all 20 Phase I sites had been activated. Amongst others, the audit gives a count of the approved, filled and vacant professional nurse posts for the ART programme, as well as nurses trained for ART, at each facility at the time.

2. The deployment, training and integration of new staff, and adaptations in task distribution through baseline and follow-up facility appraisals conducted in the first 20 (Phase I) ART sites. The baseline appraisal was conducted about one month prior to the introduction of the programme, and thereafter at two follow-up occasions: the first seven months after going operational, and the second a year thereafter.

3. The number of approved, filled and vacant nurse posts per district and per facility at the 38 Phase I and II ART sites, extracted from the official government personnel database (PERSAL). These data were obtained twice, in March and August 2006. In November 2006, additional PERSAL data were retrieved on new appointments, promotions and lateral transfers of professional nurses servicing the ART programme.

4. The training of professional nurses and other staff in ART during 2004, 2005 and 2006, obtained from the Directorate: Staff Development of the Free State Department of Health.

The findings reported here apply to a specific province and to the ART programme as implemented in the facilities of this province at a particular point in time. They are thus not necessarily generalisable to other provinces of South Africa or other countries. However, given the shared nature of the human resource crisis across contexts and a degree of nationally determined standardisation of the ART programme, it is safe to claim that the study indeed highlights core problems and constraints in the rollout of the public ART programme in South Africa and, therefore, also furnishes important lessons for other provinces and countries.

### New staff establishments for ART and distribution of ART sites per district

The professional staffing requirements per 500 patients on ART are stipulated as follows in the Comprehensive Plan: 1 medical officer; 2 professional nurses; 1 pharmacist; 1 dietician/nutritionist; 0.5 social workers. Non-professional full-time staff amount to 5 lay counsellors/CHWs and 2 administrative clerks/data capturers [7]. The Free State's initial staff establishments provided for three professional nurses at treatment sites, three (in some cases four) at combined treatment-assessment sites, and three (in exceptional cases four) at assessment sites [12,21]. In the Free State, 60 professional nurse posts were thus added to Phase I and a further 52 to Phase II sites, thus totalling 112 (later 115, after additions to some of the larger facilities). The large majority of these newly created professional nurse posts was based at PHC facilities hosting the ART programme. Relative to both patient populations (Table 1) and national norms, professional nurse staffing for the ART programme appears rather generous at the time, especially at those facilities in predominantly rural/small-town districts (see Table 1).

**Table 1:** District characteristics, patient numbers and indicators of workload [22-25]

**District**	**Lejwele-** **putswa**	**Motheo**	**Thabo ** **Mofutsanyana**	**Xhariep**	**Fezile** ** Dabi**	**Free State**
Population size	641 391	755 521	751 450	142 601	465 958	2 756 921
Antenatal HIV prevalence (2004)	33.0%	27.6%	27.1%	21.3%	32.2%	**29.5%**

Patient numbers:						
- Enrolled	5 679	9 004	8 248	2 170	3 935	**29 036**
- Eligible	2 629	3 262	3 530	785	1 348	**11 554**
- On treatment	1 117	1 536	1 806	359	882	**5 700**

Approved nurse post: patient ratios:						
- Enrolled	1:227	1:375	1:357	1:103	1:179	**1:252**
- Eligible	1:105	1:136	1:153	1:37	1:61	**1:100**
- On treatment	1:45	1:64	1:79	1:17	1:40	**1:50**

Note: Although the cited patient numbers represent the official figures for the Free State, they are considered an undercount. A more accurate estimate of the total number of patients ever started on ART by end December 2006, was around 7105, based on the number of prescriptions dispensed by the provincial pharmaceutical services.

During Phases I and II of the programme in the Free State (2004 to 2006), provincial policy was, firstly, to open an equal number of ART sites in each district and, secondly, to create uniform staff establishments with an equal number of staff allocated to the three ART facility types. Neither of these allocation strategies was premised on need, such as population sizes and densities of the districts, HIV prevalence rates, and demand for care. As a result, highly uneven professional nurse-patient ratios, as well as highly uneven workloads for nurses servicing the programme, emerged across the province (Table 1). In some districts, staff rapidly became overloaded without changes in staff establishments, while in other districts ART staff appeared underutilized.

### Recruitment of professional nurses into ART programme posts

The creation of new posts does not necessarily translate into filled positions. The addition of personnel through actual appointments is thus a more reliable indicator of systems strengthening than the creation of posts. The filling of the new ART posts occurred against a backdrop of high general vacancy levels in the public health system. In 2003, only 59.3% (7176) of 12 104 health professional posts in the Free State were filled (i.e. a vacancy rate of 40.1%), well below the national average of 68.9% filled posts (or a 31.1% vacancy rate) [10,12,23]. By mid-2006, however, professional nurse vacancy rates in the ART programme were half (15.8%) that of the PHC system as a whole (37.1%) (Table 2). Although initial recruitment into the ART programme was difficult in some districts, by November 2006, 97 (85%) of the 115 professional nurse posts allocated to the ART programme had been filled (see Table 2).

**Table 2:** Professional nurses: trends in vacancy rates of ART posts (November 2004, to November 2006) as against all

	**ART posts**				**All PHC** **posts** ** (August ** ** 2006)**
	
**District**	**November** ** 2004**	**March** ** 2006**	**August ** **2006**	**November ** **2006**	
Lejweleputswa	36.4%	8.0%	8.7%	8.0%	25.6%
Motheo	8.3%	4.6%	4.4%	0%	45.5%
Thabo Mofutsanyana	8.3%	4.6%	8.7%	13.0%	30.6%
Xhariep	46.2%	57.1%	42.9%	38.0%	54.4%
Fezile Dabi	45.5%	13.6%	18.2%	22.7%	35.9%

**Total posts**	**28.8%** (of 59*)	**16.8% ** (of 113*)	**15.8%** (of 114*)	**15.7%** (of 115*)	**37.1%** (of 1 664)

PHC posts (August 2006), per district (PERSAL data)

Note: The numbers of professional nurses differ from the original 60 (Phase I) and 112 (Phase I and II) professional nurse posts created for the ART programme; these differences are ascribed to the creation of additional posts at some of the larger sites later during programme rollout.

At inception of the ART programme, ART posts were possibly attractive due to the novelty and considerable political and bureaucratic attention focused on the programme, nationally and provincially [20]. This resulted in 'lateral transfers' of nurses from other programmes to the ART programme, which partly explains the differences in vacancy rates between the ART sites and the PHC system as a whole. These newly created posts in many cases also provided opportunities for promotion. In July 2005, all professional nursing posts for ART were upgraded to senior level. On the one hand, this led to promotion of nurses appointed in the programme (Table 3); on the other, it attracted nurses deployed in other programmes to posts into the ART programme for prospects of promotion and better remuneration. In two predominantly urban districts (Motheo and Lejweleputswa) promotions were the highest relative to the appointments made, which partly explain the low vacancy rates in the ART programme of these two districts relative to the other three districts.

**Table 3:** Promotion of ART nurses per district, November 2006 (PERSAL data)

**District**	**Filled posts**	**Promotions (% filled posts)**
Motheo	24	17 (70.8%)
Lejweleputswa	23	15 (60.0%)
Thabo Mofutsanyana	20	6 (27.3%)
Xhariep	13	6 (28.6%)
Fezile Dabi	17	9 (40.9%)

**Total**	**97**	**53 (54.6%)**

### Sources of professional nurses recruited into ART programme and patterns of geographic mobility

The origin of professional nurses recruited into the ART programme and their geographic mobility following the introduction of the ART programme were assessed through both facility appraisals and the review of PERSAL data.

A staffing inventory in the facility appraisals found that, prior to the ART programme, a total of 147 professional nurses were employed at the 16 Phase I PHC facilities. After the introduction of the programme, i.e. at the first follow-up appraisals, this number had grown to 167 (including 40 ART nurses) and remained almost the same at the second follow-up – 165 (including 37 ART nurses). The net gain in these facilities at the second follow-up appraisal was thus only 18 professional nurses. Subtracting all the ART appointments (37) from the total number of professional nurses in these facilities, there was then a net loss of 19 professional nurses in the surrounding PHC system (i.e. the 16 PHC facilities). Given that in most of the facilities observed, the ART programme was provided in a vertical manner separate from other services, this suggests considerable displacement or drainage of professional nurses towards the ART programme.

Across the Free State as a whole, one in every five (20.6%) of the 97 professional nurses appointed to the Phase I and II ART sites were genuinely new entrants to the province's staffing establishment, i.e. nurses entering the system as new graduates, or from the private and non-governmental sectors, or from other provinces and countries. The majority of professional nurses (43.2%) moved from a neighbouring (non-ART) facility in the same district to the ART providing facility (inter-facility mobility). One-quarter (24.7%) of new recruits came from another division/programme within the same facility (intra-facility mobility). Such intra-facility flow into the ART programme was also more obvious in the two predominantly urban districts (Motheo and Lejweleputswa). Little inter-district movement of nurses took place in filling the Free State's ART posts (11.3%) (see Table 4).

**Table 4:** Sources of professional nurses for the ART programme up to November 2006 (PERSAL data)

**District**	**New** **appoint-** ** ments**	**From same** ** facility**	**From** **another ** **facility in** ** same district**	**From a** **another** ** district**	**Total** **filled** ** posts**
Lejweleputswa	2 (8.7%)	10 (43.5%)	10 (43.5%)	1 (4.3%)	23
Motheo	3 (12.5%)	8 (33.3%)	11 (45.8%)	2 (8.3%)	24
Thabo Mofutsanyana	5 (25.0%)	3 (15.0%)	10 (50.0%)	2 (10.0%)	20
Xhariep	5 (38.5%)	1 (7.7%)	4 (30.7%)	3 (23.1%)	13
Fezile Dabi	5 (29.4%)	2 (11.8%)	7 (41.2%)	3 (17.6%)	17

**Total**	**20 (20.6%)**	**24 (24.7%)**	**42 (43.2%)**	**11 (11.3%)**	**97**

### Training of staff for ART

During 2004, the Free State Department of Health developed its own training curricula on ART, and offered a series of five-day training courses for personnel working in the programme. The November 2004 staff audit showed that the majority of professional nurses appointed in the ART programme (37 of 42) had received such training in the months prior to the activation of the programme. Over and above these, an additional 23 nurses had been trained for ART at the time, thus securing a significant reserve workforce for the programme.

A review of both the contents and process of ART training in 2005 resulted in a revised and integrated training model incorporating the themes of voluntary counselling and testing (VCT), integrated management of childhood illness (IMCI), prevention of mother-to-child transmission (PMTCT) and the syndromic management of STIs. This training model entails a combination of distance-based and on-site, theoretical and practical, initial and maintenance components [12]. Training for ART has been facilitated by the presence of a well-established distance-based training infrastructure offered via iCAM (Interactive Distance Communication and Management System), a television broadcasting medium which enables the Free State Department of Health to disseminate information and communicate with health workers from a central studio in the province's capital. The system reaches 38 receiving satellite sites spread over the entire province [21].

The introduction of ART thus resulted in large-scale training of staff. The change in the mode of training to a more integrated approach in 2005 increased the numbers of staff trained for ART even further, especially professional nurses. After three years, this group represents and remained the largest category of all staff trained in ART (46.5%). Moreover, during the first three years, a significant extra pool of nurses has been trained in ART relative to the number of professional nurse posts approved for the programme – 580 as opposed to the 115 approved posts. However, a limited number of enrolled/staff nurses (two) and nursing assistants (five) were trained in ART (Table 5).

**Table 5:** Number of staff trained in ART – 2004, 2005 and 2006 [27,28]

**Staff category**	**2004**	**2005**	**2006**	***Total***
Professional nurses	127	172	281	***580***
Enrolled/staff nurses	1	1	-	***2***
Nursing assistants	-	-	5	***5***
Other health professionals	87	160	154	***401***
Non-professional staff (support staff, lay health workers, etc.)	107	69	231	***407***

***Total***	***322***	***402***	***671***	***1 395***

A more general increase in training and skills development in certain HIV-related competency areas also took place in the months following the introduction of ART: between the baseline and first follow-up appraisal, of the 147 nurses employed at the assessment sites, 38 (26%) had received additional training in STI syndromic management, 32 (22%) in post-exposure prophylaxis, 28 (19%) in VCT, and 26 (18%) in PMTCT and child nutrition/growth monitoring, respectively.

### Task shifting, delegation and substitution

To compensate for the general shortage of professional staff in the public health service, various forms of task shifting and task sharing have been observed in the Free State's ART programme. Firstly, after an initial period of relying strictly on doctors for decision-making on initiation of treatment, there is growing support – also due to the scarcity of doctors in certain areas – for a nurse-initiated and nurse-monitored model of ART at PHC facilities (a form of indirect substitution according to Dovlo [3]). Similarly, the general scarcity of pharmacists in the public sector, aggravated by the strong reliance of the ART programme on pharmaceutical services, necessitates the shifting of typical pharmacist tasks, i.e. filling and dispensing prescriptions, to the more generally available professional nurses (also a form of indirect substitution).

There is little evidence in the Free State of direct substitution of professional nurses by enrolled nurses and nursing assistants, despite national guidelines advocating the use of these cadres on a larger scale. From the onset, the post establishments for the ART programme clearly favour professional nurses, and the resultant trend is that negligent numbers of enrolled nurses and nursing assistants have been recruited into the programme or trained on ART (Table 5), despite experiences elsewhere suggesting that a number of ART programme activities are readily amenable to task shifting [26].

The appointment of additional administrative staff and the better deployment of lay health workers have been more prominent as manifestations of task shifting. These cadres are generously provided for in the ART programme. During Phases I and II of the ART programme, one administrative clerk and one data capturer were allocated to each ART facility. A total of 78 such posts were created, and their subsequent occupation shows negligible vacancy rates (less than 7%). Regarding lay health workers, the consecutive facility appraisals show increased strengthening of existing complements of lay health workers through multi-skilling and additional training. Initially, most lay health workers (82%) were trained either in lay counselling, home-based care or directly observed TB treatment; by the second follow-up the majority (85%) were trained in more than one area. In addition, lay counsellors are increasingly substituting professional nurses in providing aspects of drug readiness training on their own for ART patients, a task initially performed by professional nurses [27]. Midway into the ART programme, the participation of these lay workers was boosted by a standardised and expanded system of stipends, which amounted to nearly double the remuneration previously received and also making it possible to draw larger numbers of lay health workers into the ART programme.

## Discussion

### Strengthening the system

Since its inception, it has been the explicit aim of the Comprehensive Plan to strengthen the overall health system through the ART programme. The provision of resources through ring-fenced national conditional grants reflects the programme's preoccupation with fulfilling this mandate and protecting the health system from unnecessary additional burdens. However, as the results in this study demonstrate, the general shortage of health professionals in the public health system is not immediately amenable to buy-out. Instead, the injection of new resources and the incentive structures they make possible can have the unintended consequence of weakening the overall health system by drawing essential human resources from other programmes and facilities. In many respects the human resource dynamics of the ART programme in the Free State thus reveal both the strengths and the weaknesses of human resource development and human resource management within the South African health system more generally. These relate to strategies of recruitment, deployment and retention of staff, human resource planning, decentralization of decision-making, human resource management and supervision, the role of vertical programmes, and the focus on and investment in training. The results of the study should sensitize policy makers and managers to these distorting effects.

The ART programme established many new posts, primarily in the PHC system, and for both professional and non-professional cadres. In respect of professional nurses, who are at the core of both the PHC system and ART rollout, the filling of these new posts introduced a multi-faceted dynamic in the public health system, with potentially problematic effects on the larger provincial health system within which the programme is embedded. First, of the 97 professional nurses appointed to the Free State's programme, only one-fifth [20] were newly recruited into the system. The larger majority (more than 80%) came from within the Free State's own public health system, i.e. from within the very same facilities hosting the ART programme, from other facilities within the same district, and to a lesser extent from other districts in the province. Second, the flow of professional nurses towards the few facilities providing ART in the province was no doubt encouraged by prospects of promotion and better remuneration in the new and well-resourced ART programme. Given the high vacancy rates of professional nurse posts in provincial health facilities and persistent difficulties in filling newly vacated non-ART posts within the facilities providing ART, it is unlikely that the 77 professional nurse posts vacated elsewhere in provincial facilities in favour of the ART programme would have subsequently been filled or, due to slow administrative procedures in the public sector, speedily filled. Third, the filling of professional nurse posts in the ART programme was notably better in urban/large-town areas than in the rural/small-town areas, suggesting that there may have been a degree of flow away from rural areas and thus reinforcing national trends of migration away from underserved, poorer and rural areas [28].

### Implementing uniform staffing norms for districts and ART facilities

Despite positive strides and many achievements with ART in the Free State, it is evident that the province's human resource allocation and distribution policies for the programme were not sensitive enough to the differential patient loads in the five districts. The staffing of ART sites adopted a uniform approach across the province and its districts, irrespective of differential AIDS burdens and needs among the five districts, or the varying functions performed by different types of ART sites. As a consequence, staff-patient ratios in the programme varied greatly across the province, while the presence of doctors in treatment sites meant that professional nurses were performing tasks way below their competency levels. On the other hand, however, ART facilities in rural/small-town areas generally struggled to attract sufficient professional nurses.

### More professional nurses for the public service

Provincially the planning of the ART rollout and scale-up did not take sufficient account of the existing human resource shortages in the public sector, nor of the labour-intensive nature of the ART programme, and of the likely distorting impact of special incentives to attract staff to the ART programme on the PHC system as a whole. Although yet to translate into implementation at provincial level, there is national recognition of the need to increase the production and improve the conditions of service and retention of nurses in the public sector. One such development is the national human resources for health planning framework [11] which envisages a drastic raise in the annual production of professional nurses by more than 50% (from the recent 1896 to 3000 by 2011). It also recognises that the improvement of the conditions of service and remuneration for health professionals constitute the most urgent priority. To address this priority area, the occupational specific dispensation (OSD) was announced in 2007. In terms of the OSD, all professional nurses in the public service will be re-graded according to qualifications and years of experience, and remunerated accordingly. Significant improvements for professional nurses are thus being introduced which hold favourable prospects for retaining them in the public sector. However, the same common pool of professional nurses feeding the public sector also feeds the private and non-governmental sectors and one can, therefore, expect that competitive countermeasures will be instated to secure a sufficient professional nurse workforce for the private sector's local and overseas enterprises.

A second development, commencing in 2008, is the extension of compulsory community service (CS) to professional nurses on completion of their training – a system already in place for other health professionals. About 2000 new nursing graduates will be deployed annually for one year in the public health system, especially in under-resourced areas. For the Free State this ensures an additional 65 professional nurses in the public service in 2008, and similar numbers to follow in years to come. This could compensate for some of the demands created by the ART programme.

### Integration, decentralization and centralization of service delivery

Weaknesses in the planning and deployment functions in the ART programme are to some extent a product of the over-centralized and vertical nature of the ART programme. Minimum staff establishments for the ART programme were decided nationally and facilities required national accreditation before they could dispense ART to patients. This promoted an over-rigid design that left little room for provincial and even less for district-level innovation and adaptation. In addition, the ART programme was overly doctor- and pharmacist-centred. One of the effects was that within ART sites, the programme was implemented in a vertical manner with staff reserved for the ART programme only, even if these were at times under-utilised [20]. To counter these deficiencies, three notable human resource-related strategies have in time emerged to alleviate burdens, spread workloads more evenly, and address the general scarcity of professional nurses in the province. Firstly, and promoted by the broad-based training offered in the province, steps are being taken to initiate greater service and staff integration by rotating staff between programmes offered in the ART-hosting facility and thus spreading workload more equally within these PHC facilities. Secondly, decentralization of ART services to peripheral facilities not providing ART is being promoted with a view to spread the workload more equally among different facilities. Both such integration and decentralization hold promise for greater access and greater equity for patients entering the ART programme. With less central control, both these distributive mechanisms should be in the decision-making space of health managers at the district and facility levels, and both are likely to prove effective in alleviating the pressure on professional nurses in poor-resourced settings. Thirdly, centralization of service components of the ART programme has also proven to be useful in light of shortfalls of human resources of a specific kind. For example, in an attempt to counteract the general shortages of pharmacists in rural areas, a centralized ARV dispensary has been established in the Free State's capital to service a large rural district from a central, well resourced point. This initiative may also alleviate professional nurses of a dispensing workload.

### Task shifting and sharing

After the initial prevalence of a doctor-centric model and heavy reliance on pharmacists to deliver ART, the provision of ART is increasingly shifted onto professional nurses at PHC facilities, i.e. nurses perform the typical tasks of doctors and pharmacists respectively, either by initiating treatment and prescribing ARV drugs, or by filling and dispensing prescriptions. In broader context, such task shifting initiatives will remain limited, unless more significant progress is made with redefining the scopes of the professions and scopes of practices to allow for the optimum use of human resources.

The shifting of tasks from professional nurses to community health workers and administrative support staff to relieve nurses of non-clinical tasks and unwarranted workload is quite pronounced and perhaps the best developed form of delegation and substitution in the ART programme in the Free State. Major strides have been made in both forms of delegation by adding significant numbers of these cadres to the staff establishments of ART sites.

Of concern, however, is the negligent utilization of semi-professional or mid-level nursing staff in the Free State's ART programme, despite the fact that this capacity is widely employed in the ART programmes in human resource-constrained settings, including other provinces in South Africa, but even more so in other African countries. Furthermore, this capacity remains largely unexplored in the province, despite national guidelines providing generously for the use of these lower level nurses in the ART programme: totals of 1255 enrolled nurses and 1255 assistant nurses were foreseen by 2008, i.e. for both these categories a ratio of 1:1.5 professional nurses is implied [7]. This imbalance in the Free State ART programme could be ascribed to the strict adherence to the initial norms for staffing the ART sites. A similar neglect, which perpetuates the existing pattern, is observed in the low numbers of mid-level nurses trained for the ART programme in the province. There is thus ample scope for employing enrolled nurses and nursing assistants to a larger extent in the Free State's ART programme in order to alleviate the pressure on professional nurses.

### Training of staff

Strengthening of the health system is not only about more human resources. Strengthening also occurs through training and systems development, amongst others, through the upgrading of health facilities and infrastructure, laboratories, pharmacies, and IT systems. A key strength in the Free State was the rapid organisation and implementation of training on ART and related programme components, including the effective use of information technology for continuing education. In addition, the training initiatives stimulated by the ART programme also brought with it a wave of new training across the PHC care system, and going beyond the ART sites and ART themes. These factors may in part explain why nurses interviewed in the PHC system of the Free State have been more positive than negative about the ART programme, despite the additional demands made of them [29,30]. However, on its own, training is not the sole solution. It is not uncommon for in-service training to become the centre of human resource development, rather than the other more complex tasks of supply, planning and management [1,2].

## Conclusion

As is the case with other PHC programmes, professional nurses form the backbone of the ART programme in South Africa. In the absence of sufficient numbers of other types of health professionals – doctors, pharmacists, dieticians and social workers – the onus is increasingly shifted onto the widely available professional nurses to fill emerging service gaps. However, in the long term, without concerted efforts to increase the supply of professional nurses and other health workers, neither goals of the Comprehensive Plan – universal access to ART and strengthening the health system – will be achieved. However, more than mere numbers of professional nurses and support staff for nurses are necessary for meeting the human resource challenges posed by the HIV/AIDS epidemic and the scale-up of ART. Equally important is the effective supervision, management and support of staff, better conditions of service and remuneration with a view to heightened work satisfaction, retention and productivity. In most recent years, several valuable strategies and tools have been developed with a view to achieve these human resource-related goals [1,2]. However, in the face of persistent shortages of professional health personnel and the growing pressures on them to deliver the needed services, it is imperative that the vital and increasing role of patients and community members in scale-up operations should be optimally explored and employed [1].

To address the spectrum of human resource shortages and skills deficits in the sphere of ART programmes (but also in the health domain at large), policy makers and managers in heavy-burdened countries have to devise new strategies and even have to make paradigm shifts to cope with the challenges at regional, national, sub-national and facility levels, either by exploring new human capacity or by utilising and managing the existing, meagre pools of personnel better [4,17,18]. Much has indeed been accomplished in this direction with the ART programme in the Free State. However, it is also clear that much more new thinking is necessary to overcome the growing human capacity deficits in the light of the ever growing demands made on human resources for HIV care.

Regarding further research, the study illustrates the value of recording information on the dynamics of human resources in general and as far as a specific programme is concerned. The study indeed gives an idea of what kind of data is important for research of this nature; at the same time it also shows what is lacking in the data presented, e.g. information on what happened to the vacancies generated by the movement of nurses to ART facilities.

## Competing interests

The authors declare that they have no competing interests.

## Authors' contributions

DvR and FS conceptualised the research, managed data collection and wrote the article. HS provided inputs into the design, interpretation and write-up phases. LL extracted, compiled and made available the relevant provincial data on human resources. All authors read and approved the final manuscript.
